# COVID-19, Eosinophils, and Biologicals for Severe Asthma

**DOI:** 10.3389/falgy.2022.859376

**Published:** 2022-04-28

**Authors:** Carlo Lombardi, Diego Bagnasco, Giovanni Passalacqua

**Affiliations:** ^1^Departmental Unit of Allergology, Clinical Immunology and Pneumology, Istituto Ospedaliero Fondazione Poliambulanza, Brescia, Italy; ^2^Allergy and Respiratory Diseases, IRCCS Policlinico S.Martino-University of Genoa, Genoa, Italy

**Keywords:** biologicals, COVID-19, eosinophils, outcome, severe asthma

## Introduction

The current literature shows that many hospitalized patients with documented COVID-19 disease have eosinopenia. Thus, the peripheral blood eosinophil count could be regarded as a possible biomarker for evaluation and prognosis (1). In particular, eosinopenia seems to be an indicator of severity among patients with COVID-19, whereas an increasing eosinophil count is associated with a better prognosis during COVID-19 disease, including a lower incidence of complications and mortality (2). On these premises, it can be hypothesized that eosinophils have, to some extent, the ability to attenuate viral replication and protect against the development of the uncontrolled inflammatory response underlying the severe COVID-19 disease.

According to the available literature, asthma seems not to represent a relevant risk factor for COVID-19 infection or a predictor of the worst clinical course. However, its real contribution to the overall risk may also depend on the presence of environmental and behavioral factors (i.e., smoking), type and severity of asthma (i.e., non-type 2 asthma phenotypes), adherence to therapy, and comorbidities (3). It was also hypothesized that asthmatics with Type 2 phenotype, which usually includes an increased peripheral blood eosinophil count, would have a more favorable outcome (4). Furthermore, it has been proposed that inhaled corticosteroids (ICS) may confer some degree of protection against severe acute respiratory syndrome coronavirus 2 (SARS-CoV-2) infection and the development of severe disease by reducing the expression of angiotensin-converting enzyme-2 and transmembrane protease serine in the lung (5). On the other hand, this raises concerns about the use of biologicals in severe asthmatic patients, namely interleukin 5 (IL-5) antagonists, anti-immunoglobulin E (anti-IgE), and anti-IL-4/IL-13. They are able to modulate, decrease or deplete circulating eosinophils, and thus a detrimental effect in COVID-19 disease could be expected. Furthermore, eosinophils express a broad range of pattern-recognition receptors, including toll-like receptors (TLRs), nucleotide-binding oligomerization domain-like receptors, retinoic acid-inducible gene-like receptors, C-type lectin receptors, and a receptor for advanced glycation end products, which supports their potential role in responses against pathogen-associated molecular patterns induced by viral, bacterial, and fungal infections (6). Indeed, an increasing number of recent observations indicate that eosinophils are not only associated with the pathogenesis of a wide range of diseases but also contribute to the maintenance of homeostatic responses (7).

To assess the extent of such phenomenon, we carried out a survey as a narrative review using the main search engines on the studies that have addressed this issue. We identified 15 studies on the use of biological agents in severe asthma in the COVID-19 era (Table 1), including a total of 98 patients with severe asthma and concomitant COVID-19 disease, who were receiving omalizumab (30 patients), mepolizumab (32), benralizumab (18), reslizumab (4) and dupilumab (4), plus 10 patients receiving unspecified IL-5 antagonists. As summarized in Table 1, the clinical course of patients was overall favorable. Among 98 patients, 28 (29%) were hospitalized, and 8 (8%) were in intensive care. There were three deaths (3%). According to the literature, all those patients had comorbidities variably associated with asthma (diabetes, obesity, and systemic hypertension). Analyzing the data of the studies summarized in Table 1, it appears that the incidence of severe events (including deaths) was not greater in patients treated with biologicals, as compared with others that did not receive them. Looking more into details of literature, the observation suggests that the more severe adverse events were more probably determined by comorbidities (8, 9), rather than by the biological therapy. It should also be considered that therapy with mepolizumab does not result in complete suppression of blood and bone marrow eosinophil levels (10) and that the use of dupilumab, at least in the initial period, may be associated with transient blood eosinophilia (11): both of these factors may lead to a protective effect by eosinophils, as might occur in the context of SARS-CoV-2 infection.

**Table 1:** Reports on patients receiving biologicals and having COVID-19.

**Author (year)**	**Reference**	**Pats**	**Treatment**	**Outcome**
Aksu K (2021)	Allergy Asthma Proc. 2021; 42: e55-e57	1	Mepolizumab	Severe asthma not worsened by COVID-19; favorable outcome
Azim (2021)	Ann Allergy Asthma Immunol. 2021; 126:438-40	4	Mepolizumab	Only 1 patient required hospitalization and respiratory support, and already had risk factors for COVID19
Matucci A (2021)	Allergy 2021; 76: 871-874	3; 1	Omalizumab; Benralizumab	2 cases of non-serious COVID-19, 2 cases of severe and critical COVID (Omalizumab), no death
Eger K (2020)	Respir Med. 2020 24;177:106287	2; 1; 3; 1; 2	Omalizumab; Dupilumab; Mepolizumab; Reslizumab; Benralizumab	7 cases required hospitalization, of which 5 with intubation in intensive care. 1 death
Tanabe N (2021)	Allergol Int. 2021; 70: 274-76	1	Dupilumab	The patient was admitted in intensive care due to COVID-19. Favorable outcome.
Bhalla A (2021)	Allergy. 2021;76:957-958	1	Dupilumab	Uncontrolled asthma, 9 weeks COVID-19 infection
Förster-Ruhrmann U (2020)	J Allergy Clin Immunol. 2020; 146 218-220	1	Dupilumab	Chronic rhinosinusitis with nasal polyps (CRSwNP) + severe asthma; mild COVID-19 with full recovery
Rial MJ (2021).	J Allergy Clin Immunol Pract.2021;9: 487-489	14; 11; 3; 7	Omalizumab Mepolizumab, Reslizumab Benralizumab	8/35 cases required hospitalization, 1 (Omalizumab) admitted to intensive care, 1 (82 yrs) died as a result of complications due to COVID19 and presence of comorbidities
Renner A (2020)	J Asthma. 2020; 18: 1-3	1	Benralizumab	Reduced asthma control during COVID-19 infection
Renner A (2020)	ERJ Open Res. 2020;6: 00457-2020)	2	Benralizumab	Asthma control unchanged during, before and after COVID-19 infection
Kroes JA (2021)	Eur J Hosp Pharm. 2021; ejhpharm-2020-002660)	1	Benralizumab	After admission, benralizumab was discontinued and severe bronchial obstruction developed. After the next administration of benralizumab no further symptoms developed.
Heffler E 2021	Allergy 2021; 76: 887-892	6; 13; 2	Omalizumab Mepolizumab Benralizumab	Four patients were hospitalized, one of which in ICU; among hospitalized patients, 1 death with comorbidities.
García-Moguel I (2020)	Ann Allergy Asthma Immunol. 2020; 125: 357-359	2	Benralizumab	severe asthma, mild COVID-19
Hanon S (2020)	Eur Respir J. 2020; 56: 2002857	4; 10	Omalizumab; Anti IL-5 (not specified)	Only 5 hospitalized (with a short hospital stay)
Lommatzsch M (2020)	Allergy 2020; 75:2705-2708)	1	Omalizumab	No evidence of asthma exacerbation, loss of asthma control or pneumonia during COVID infection
TOTAL		**98**	32 Mepolizumab; 30 Omalizumab; 18 Benralizumab; 4 Reslizumab; 4 Dupilumab	

In conclusion, this report found that patients with severe asthma requiring a biologic and COVID-19 infection do not have a more relevant disease severity and mortality. International documents recommend continuing the standard asthma therapies, including inhaled steroids and biological agents. Notably, the use of inhaled steroids does not increase the risks. Eventually, the decision to continue or postpone biologic therapy in patients already infected with SARS-CoV-2 should be individualized (12). Considering the increasing number of patients with severe asthma treated with biological agents, the data appear overall reassuring (13). Biological inhibitors of type 2 response can have a possible impact on aberrant immune response, and thus can protect infected subjects from severe complications of COVID-19 (14).

Nonetheless, it appears important to assess if the pharmacologically-induced reduced function/number of eosinophils by biological agents may represent harm. As repeatedly suggested, it is also important to continue the biological treatment in severe asthma patients, with special attention to comorbidities.

## Data Availability Statement

The original contributions presented in the study are included in the article/supplementary material, further inquiries can be directed to the corresponding author.

## Author Contributions

CL contributed to conception and design of the study, organized the database, and wrote the first draft of the manuscript. GP and DB wrote sections of the manuscript. All authors contributed to manuscript revision, read, and approved the submitted version.

## Conflict of Interest

The authors declare that the research was conducted in the absence of any commercial or financial relationships that could be construed as a potential conflict of interest.

## Publisher's Note

All claims expressed in this article are solely those of the authors and do not necessarily represent those of their affiliated organizations, or those of the publisher, the editors and the reviewers. Any product that may be evaluated in this article, or claim that may be made by its manufacturer, is not guaranteed or endorsed by the publisher.
